# Environmental and parental risk factors for congenital solitary functioning kidney — a case–control study

**DOI:** 10.1007/s00467-023-05900-6

**Published:** 2023-02-20

**Authors:** Sander Groen in ‘t Woud, Nel Roeleveld, Iris A. L. M. van Rooij, Wout F. J. Feitz, Michiel F. Schreuder, Loes F. M. van der Zanden, for the SOFIA study group, J. A. E. van Wijk, R. Westland, K. Y. Renkema, M. R. Lilien, M. G. Keijzer-Veen, F. J. Kloosterman, M. G. Steffens, V. Gracchi, B. Zegers, P. E. Jira, H. van der Deure, R. W. G. van Rooij, E. Wijnands - van den Berg, M. Breukels, S. M. H. B. de Pont, E. Harnisch, C. M. L. van Dael, D. Creemers, R. de Moor, A. Y. Konijnenberg, E. Knots, E. C. van der Kuur, M. J. Jacobs, M. Koppejan-Stapel, A. Pijning, E. Dorresteijn, R. W. J. Leunissen, R. Rijlaarsdam, R. del Canho, B. Semmekrot, A. Dings-Lammertink, I. J. M. Nijhuis, M. J. van Ledden-Klok, L. M. van den Broek, C. Meine Jansen, M. C. G. Beeren, H. E. Blokland-Loggers, C. Dorrepaal, L. J. W. M. Pierik, A. L. Tanja

**Affiliations:** 1 Department for Health Evidence, Radboud University Medical Center, P.O. Box 9101, 6500 HB Nijmegen, The Netherlands; 2 Department of Pediatric Nephrology, Radboudumc Amalia Children’s Hospital, Nijmegen, The Netherlands; 3 Department of Urology, Division of Pediatric Urology, Radboudumc Amalia Children’s Hospital, Nijmegen, The Netherlands

**Keywords:** Solitary functioning kidney, Congenital anomalies of the kidney and urinary tract, Environmental risk factors, Etiology, Kidney development

## Abstract

**Background:**

The etiology of congenital solitary functioning kidney (CSFK) is largely unknown but likely includes various risk factors. We performed a case–control study to compare exposure to environmental and parental risk factors during embryonic kidney development between children with CSFK and healthy controls.

**Methods:**

We included 434 children with CSFK and 1302 healthy controls from the AGORA data- and biobank matched on year of birth. Exposure to potential risk factors was investigated using parental questionnaire data. Crude and adjusted odds ratios (aORs) with 95% confidence intervals (CIs) were estimated for each potential risk factor. Multiple imputation was used to deal with missing values. Confounders for each potential risk factor were selected using directed acyclic graphs.

**Results:**

Maternal stress was newly identified as a risk factor for CSFK (aOR 2.1, 95% CI 1.2–3.5). Known associations with conception using in vitro fertilization/intracytoplasmic sperm injection (aOR 1.8, 95% CI 1.0–3.2), maternal infections during pregnancy (aOR 2.5, 95% CI 1.4–4.7), smoking during pregnancy (aOR 1.4, 95% CI 1.0–2.0), and parental CAKUT (aOR 6.6, 95% CI 2.9–15.1) were confirmed, but previous associations with diabetes and obesity could not be replicated. Folic acid supplement use and younger maternal age seemed to reduce the risk of CSFK (aORs 0.7, 95% CI 0.5–1.0, and 0.8, 95% CI 0.6–1.0, respectively).

**Conclusions:**

Environmental and parental risk factors are likely to be involved in the development of CSFK and future studies should combine genetic, environmental, and gene-environment interaction analyses. Women wanting to become pregnant should consider optimizing their health and lifestyle.

**Graphical abstract:**

**Figure:**
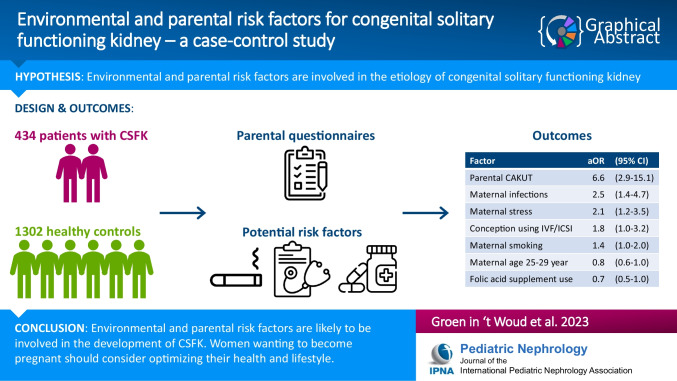
A higher-resolution version of the Graphical abstract is available as Supplementary information

**Supplementary Information:**

The online version contains supplementary material available at 10.1007/s00467-023-05900-6.

## Introduction

Human kidney development is a process that can be divided into three stages: in the third week after conception, the pronephros is formed, which regresses and is replaced by the mesonephros from week 4. Development of the final kidney (metanephros) starts in week 5 and formation of the kidney is complete around gestational week 36 [1]. A series of crucial events takes place in week 5, starting with the appearance of the ureteric bud on day 28 followed by an invasion of the metanephric mesenchyme on day 32 [1]. When either of these processes is disrupted, agenesis of the kidney will occur [2]. After the invasion of the metanephric mesenchyme, the ureteric bud starts to bifurcate, with each branch subsequently bifurcating in a repetitive process known as branching morphogenesis, which forms the collecting system [1]. Disturbances in branching morphogenesis may result in multicystic kidney dysplasia (MCDK) [3].

Unilateral kidney agenesis (UKA) and MCDK are the most common causes of a congenital solitary functioning kidney (CSFK), which is a birth defect with an estimated prevalence of 1 in 1500 live births [4]. Based on the results of an animal model, children with CSFK only have an estimated 70% of the number of nephrons of individuals with two kidneys [5], which results in an increased risk of kidney injury and necessitates long-term follow-up [6]. Despite the increased use of genetic screening, a monogenic cause for the CSFK can be found in only 10–20% of patients [7]. Therefore, the etiology is thought to be multifactorial, with both genetic and environmental factors involved [7].

Several studies have investigated environmental risk factors for CSFK, often as part of cohorts of patients with other congenital anomalies of the kidney and urinary tract (CAKUT) and sometimes stratified by specific anomalies. Previously reported risk factors for CSFK specifically include maternal diabetes [8–10], obesity [10], alcohol use [9, 11], and both younger and older age [9]. Other factors, such as maternal smoking [12], infections during pregnancy [13, 14], and use of assisted reproductive technologies [15, 16], increase the risk of CAKUT but were not studied for CSFK separately. Results for folic acid supplementation vary, with some studies showing a protective effect on CAKUT [17, 18], while others found no effect [19], and our group reported an increased risk in a previous study [16].

Studies investigating environmental causes of birth defects are important, since knowledge about these causes may lead to improved preventive efforts and can help answer the causality questions that parents of children with birth defects often have. Many challenges are present when conducting such studies, however, including the classification of birth defects, exposure assessment, correction for confounders, and dealing with small numbers and missing values. To fill the paucity of knowledge in the etiology of CSFK and overcome some of these challenges, we created a large database with detailed information on both the clinical phenotype and exposures to potential environmental and parental risk factors for CSFK cases and healthy controls. We performed a case–control study to get more insight into the role of these risk factors in the etiology of CSFK.

## Methods

### Study participants

**Fig. 1:**
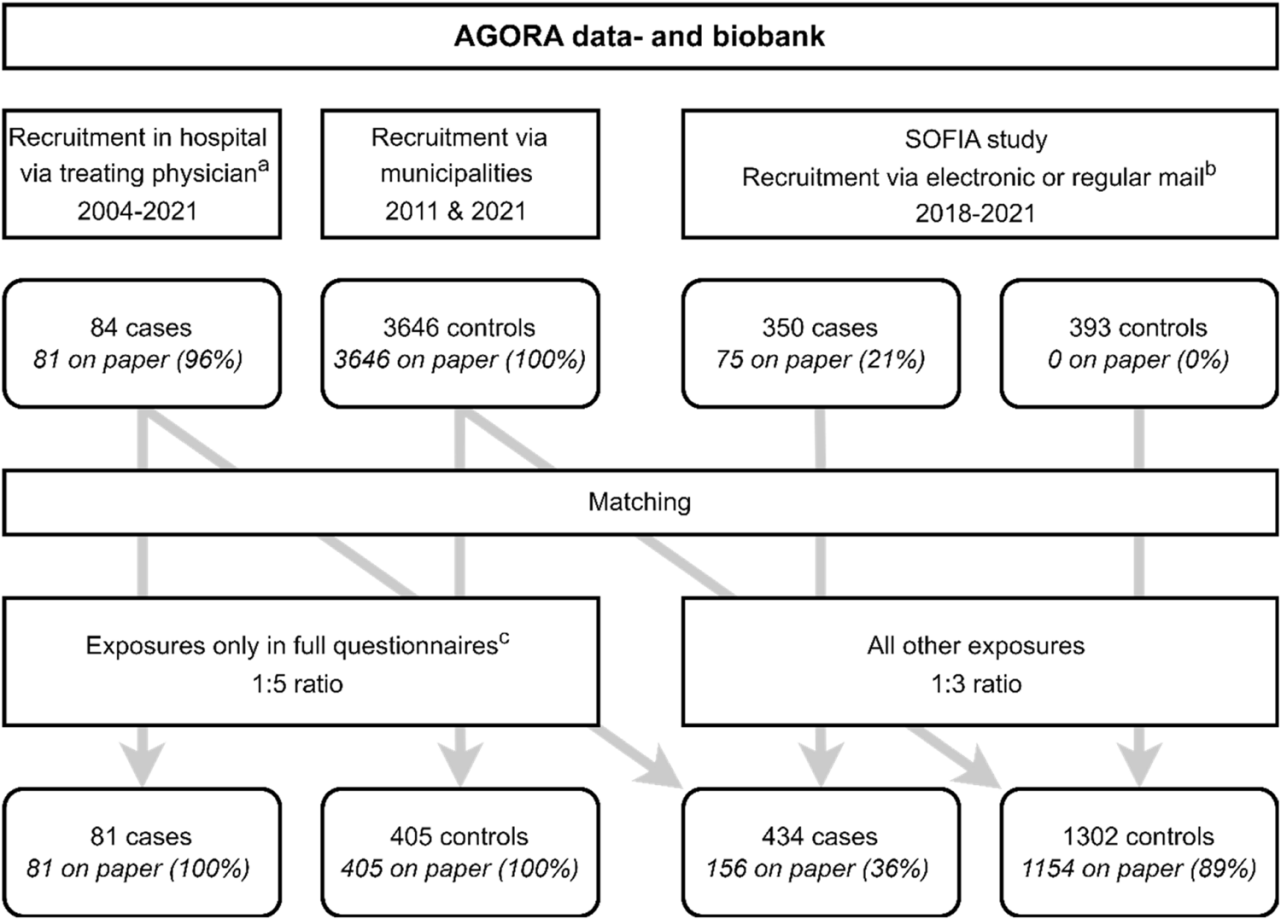
Flowchart of recruitment and selection of cases and controls. ^a^Recruitment via treating physician was performed in the Radboud university medical center (2004–2021) and University Medical Center Utrecht (2013–2021). ^b^Recruitment for the SOFIA study was performed via 36 Dutch hospitals, including the Radboud university medical center and University Medical Center Utrecht. Only patients not yet included in the AGORA data- and biobank were invited to participate. ^c^Exposures only in full questionnaires were infection, diet, and stress

The AGORA (Aetiologic research into Genetic and Occupational/environmental Risk factors for Anomalies in children) data- and biobank contain clinical information, DNA samples, and questionnaire data for patients with birth defects [20]. Parents of patients with a birth defect visiting the Radboud university medical center (from 2004) or University Medical Center (UMC) Utrecht (from 2013) were asked to participate in AGORA and received a paper questionnaire regarding environmental, parental, and other relevant exposures before and during the pregnancy of their child. To recruit healthy controls for the AGORA data- and biobank, 39 Dutch municipalities provided a random sample of children born between 1990 and 2010 in 2011. Parents of the selected children were invited to participate in the AGORA data- and biobank by filling out the same questionnaire as the parents of patients. In 2021, a similar approach was used to obtain data for children born from 2011 through 2021. All patients with CSFK, defined as a solitary functioning kidney resulting from UKA or MCDK, and part of the controls were included in the current study (see Fig. 1).

As the number of patients with CSFK in the AGORA data- and biobank was limited, the SOFIA (SOlitary Functioning kIdney: Aetiology and prognosis) study was initiated in 2018, with the aim to study the etiology and prognosis of patients with SFK. For this study, which was embedded in AGORA, additional CSFK patients were recruited in 36 hospitals in The Netherlands, including the Radboud university medical center and UMC Utrecht [6]. Parents of participating patients previously treated in these hospitals received a shortened version of the original AGORA questionnaire and could choose between online or paper completion. They were also asked to invite unrelated parents of healthy children to fill out this shortened questionnaire online.

For the current analyses, all patients with CSFK born between January 1st, 1993, and December 31st, 2020, diagnosed with UKA or MCDK before their 18th birthday, and with a completed original or shortened AGORA questionnaire were included. Children without major birth defects (defined using EUROCAT guidelines [21]) recruited via municipalities in 2011 or 2021 or via parents of children included in the SOFIA study were eligible to serve as healthy controls. The AGORA data- and biobank and the SOFIA study were approved by the Regional Committee on Research Involving Human Subjects.

### Risk factors

A selection of relevant questions from the AGORA questionnaire (available in Supplementary Material) was used to assess exposure to the risk factors under study. We considered the following potential risk factors for CSFK: family history of CAKUT (defined as maternal and/or paternal CAKUT), season of conception, parental age at conception, and parental subfertility and/or conception using artificial reproductive technologies (ART). In addition, the following maternal risk factors were studied: gravidity, body mass index (BMI) at conception, preexisting or gestational diabetes mellitus (DM), preexisting hypertension, infections during pregnancy, medication use during pregnancy, smoking, alcohol consumption, use of folic acid or folic acid-containing multivitamins, diet, and self-reported stress during pregnancy.

Maternal and paternal age were studied as both continuous and categorized (< 25, 25–29, 30–34, 35–39, ≥ 40 years) variables. Couples were considered subfertile if one of the parents was ever diagnosed with subfertility by a doctor or if the time to conception was > 12 months [22]. We grouped ARTs hierarchically into conception using intra-uterine insemination only (IUI), hormonal treatment, or in-vitro fertilization (IVF)/intracytoplasmic sperm injection (ICSI). Gravidity was categorized into first or subsequent pregnancy. Body mass index was classified as underweight (< 18.5 kg/m^2^), normal (18.5–24.9 kg/m^2^), overweight (25–29.9 kg/m^2^), or obese (≥ 30 kg/m^2^). Diabetes was considered preexisting when reported from the start of pregnancy and gestational when first diagnosed during the index pregnancy. Preexisting hypertension was any hypertension discovered before the 20th week of gestation, in accordance with guidelines from the American College of Obstetricians and Gynecologists [23]. Maternal infections during pregnancy were divided into cystitis and other episodes of fever or infection. Medications of interest included diabetes medications (including insulin), antihypertensive medications, inhalation corticosteroids, and anti-epileptic drugs. Maternal smoking and alcohol use were divided into three exposure groups: exposed during the etiologically relevant period (defined as exposure from the 6th week of pregnancy onwards), exposed but not in the etiologically relevant period (defined as exposure in the 3 months before and/or during the first 5 weeks of pregnancy), and not exposed at all in the 3 months before or during pregnancy. Similarly, the use of folic acid supplements or folic acid-containing multivitamins was classified in a three-level variable: “use as recommended” for women who used folic acid supplementation during the entire advised period (initiation before pregnancy and continued through at least the 8th week of pregnancy), “suboptimal use” (only part of the advised period), or no use during the advised period. Sensitivity analyses were performed in which only prenatal multivitamins were taken into account. Lastly, maternal diets (vegetarian or low-salt) and perceived psychological stress were investigated as potential risk factors. All exposure data were self-reported.

Detailed questions regarding infection, diet, and stress were only asked in the original questionnaire, but not in the shortened version. Therefore, analysis of these factors was limited to subjects filling out the original questionnaire. Since mothers carrying a child with CSFK might indicate higher levels of stress in late pregnancy, resulting in reversed causation, we also performed analyses while only including women who reported stress in the first trimester. Year of childbirth, maternal ethnicity (European or other), and maternal education level (low, medium, or high) were considered potential confounders but not potential risk factors.

### Statistical analyses

Each case was matched to three controls born in the same year to account for differences in the year of childbirth between cases and controls. For the analyses limited to cases and controls that filled out the original questionnaires, we used a 5:1 ratio for additional power. Crude odds ratios (ORs) with 95% confidence intervals (CIs) were estimated for all risk factors mentioned above using conditional logistic regression. To facilitate analyses of all participants, even in case of missing data, multiple imputation was used. Ten imputed datasets were created in which missing values were imputed using the information of all variables listed as potential risk factors, as well as the potential confounders and the time between birth and filling out the questionnaire. Multiple imputation was performed using the Markov Chain Monte Carlo method with predictive mean matching, under the assumption that data was missing at random. Adjusted ORs were estimated using conditional logistic regression corrected for minimally sufficient sets of confounders for each potential risk factor separately, derived from directed acyclic graphs (DAGs, available in supplementary material) created in DAGitty [24]. We used DAGs, because this method requires making assumptions about causal associations between variables explicitly [25]. Moreover, it allows for the selection of minimally needed sets of confounders, which increases statistical efficiency and avoids overcorrection introducing bias [25]. Adjusted ORs were not estimated when less than five cases were exposed. Matching, data imputation, and statistical analysis were performed using IBM SPSS statistics version 25.0.

## Results

The total study population consisted of 434 CSFK patients and 4039 healthy controls for whom completed questionnaires were available. The cause of the CSFK was UKA in 151 patients and MCDK in 283 patients (detailed clinical description provided in Supplementary Table 1 and [6]). Of the 4039 controls, 1302 were matched to cases for the main analyses and 405 controls were matched for the analyses limited to the original questionnaire (Fig. 1). Part of the study population (40 patients and 677 controls) was included in a previous study by our group [16]. Table 1 shows that matching eliminated any differences in the year of childbirth, with less than 10% of the study population born before 2000. Patients and controls were also comparable with respect to maternal self-reported ethnic background, but mothers of controls were slightly higher educated and more likely to have completed the questionnaire on paper. The median time between childbirth and completion of the questionnaire was slightly shorter in controls. Only 3% of the questionnaires were filled out by the father.

**Table 1:** Characteristics of patients with congenital solitary functioning kidneys as well as healthy population-based controls matched on year of childbirth

	Controls (*n* = 1302)	Cases (*n* = 434)
Year of childbirth		
< 2000	111 (9%)	37 (9%)
2000–2004	168 (13%)	56 (13%)
2005–2009	318 (24%)	106 (24%)
2010–2014	390 (30%)	130 (30%)
≥ 2015	315 (24%)	105 (24%)
Maternal ethnicity		
European	1216 (93%)	406 (94%)
Other	71 (6%)	26 (6%)
Missing	15 (1%)	2 (1%)
Maternal education level		
Low	100 (8%)	53 (12%)
Intermediate	472 (36%)	161 (37%)
High	712 (55%)	218 (50%)
Missing	18 (1%)	2 (1%)
Type of questionnaire		
Paper	1154 (89%)	156 (36%)
Online	148 (11%)	278 (64%)
Time to completion^a^		
Years	6.2 (3.7–8.8)	6.9 (2.8–11.8)

^a^Time between the date of childbirth and the date of filling out the questionnaire (median, interquartile range)

Several factors were associated with CSFK development in both univariable and multivariable analyses (Table 2). The strongest risk factor was a family history of CAKUT (aOR 6.6, 95% CI 2.9–15.1), while other risk factors included maternal infections during pregnancy (aOR 2.5, 95% CI 1.4–4.7), maternal stress (aOR 2.1, 95% CI 1.2–3.5), and conception after IVF/ICSI (aOR 1.8, 95% CI 1.0–3.2). Smoking during the etiological period seemed to be associated with an increased risk of CSFK as well (aOR 1.4, 95% CI 1.0–2.0), whereas a decreased OR was observed for alcohol use outside the etiological period (aOR 0.7, 95% CI 0.6–0.9). Other factors presumably associated with a decreased risk of CSFK included the correct use of folic acid supplementation (aOR 0.7, 95% CI 0.5–1.0) and a young maternal age (25–30 years, compared to 30–35 years; aOR 0.8, 95% CI 0.6–1.0). A high maternal BMI was not associated with CSFK development, but a low maternal BMI pointed in the direction of an increased risk (aOR 1.6, 95% CI 0.9–2.9). Our study showed no evidence of an effect of gravidity, season of conception, preexisting or gestational diabetes, preexisting hypertension, and paternal age. The low numbers of exposed participants prevented us from drawing conclusions for medication use and maternal diet.

**Table 2:** Crude and adjusted odds ratios for the associations between potential risk factors and congenital solitary functioning kidney

	Controls (***n*** = 1302)	Cases (***n*** = 434)	cOR	aOR^a^	95% CI low	95% CI high
Gravidity						
First pregnancy	550 (42%)	179 (41%)	1.0	1.0	ref	ref
Subsequent pregnancy	716 (55%)	236 (54%)	1.0	1.0	0.8	1.2
Missing	36 (3%)	19 (4%)	-	-	-	-
Season of conception						
Spring	321 (25%)	98 (23%)	1.0	1.0	ref	ref
Summer	305 (23%)	112 (26%)	1.2	1.2	0.9	1.6
Fall	335 (26%)	115 (27%)	1.1	1.1	0.8	1.5
Winter	327 (25%)	100 (23%)	1.0	1.0	0.7	1.4
Missing	14 (1%)	9 (2%)	-	-	-	-
Maternal age						
≤ 24 year	57 (4%)	25 (6%)	1.2	1.2	0.6	2.2
25–29 year	388 (30%)	108 (25%)	**0.8**	**0.8**	**0.6**	**1.0**
30–34 year	558 (43%)	195 (45%)	1.0	1.0	ref	ref
35–39 year	243 (19%)	89 (21%)	1.0	1.1	0.8	1.5
≥ 40 year	36 (3%)	6 (1%)	0.5	0.5	0.2	1.3
Missing	20 (2%)	11 (3%)	-	-	-	-
Maternal BMI						
Underweight (< 18.5 kg/m^2^)	37 (3%)	21 (5%)	1.8	1.6	0.9	2.9
Normal (18.5–24.9 kg/m^2^)	857 (66%)	270 (62%)	1.0	1.0	ref	ref
Overweight (25–29.9 kg/m^2^)	258 (20%)	93 (21%)	1.2	1.1	0.9	1.5
Obese (≥ 30 kg/m^2^)	90 (7%)	35 (8%)	1.2	1.1	0.7	1.8
Missing	60 (5%)	15 (4%)	-	-	-	-
Subfertility						
Fertile	947 (80%)	337 (80%)	1.0	1.0	ref	ref
Subfertile without ART	149 (13%)	53 (13%)	0.9	1.0	0.7	1.4
IUI without hormones	16 (1%)	5 (1%)	0.9	0.8	0.3	2.4
Hormonal without IVF/ICSI	36 (3%)	9 (2%)	0.7	0.7	0.3	1.5
IVF/ICSI	33 (3%)	20 (5%)	**1.7**	**1.8**	**1.0**	**3.2**
Maternal diabetes						
No diabetes	1248 (96%)	416 (96%)	1.0	1.0	ref	ref
Preexisting diabetes	4 (0%)	1 (0%)	0.8	^	^	^
Gestational diabetes	32 (3%)	12 (3%)	1.1	1.1	0.5	2.2
Missing	18 (1%)	5 (1%)	-	-	-	-
Preexisting hypertension						
No	1259 (97%)	423 (98%)	1.0	1.0	ref	ref
Yes	22 (2%)	6 (1%)	0.8	0.7	0.3	1.8
Missing	21 (2%)	5 (1%)	-	-	-	-
Maternal infections*						
No infection	314 (78%)	52 (64%)	1.0	1.0	ref	ref
Cystitis	37 (9%)	9 (11%)	1.4	1.5	0.6	3.6
Other infection/fever	52 (13%)	23 (27%)	**2.6**	**2.5**	**1.4**	**4.7**
Missing	8 (2%)	0 (0%)	-	-	-	-
Anti-diabetic medication						
No	1265 (97%)	426 (98%)	1.0	1.0	ref	ref
Yes	10 (1%)	5 (1%)	1.5	1.7	0.4	7.6
Missing	27 (2%)	3 (1%)	-	-	-	-
Anti-hypertensive medication						
No	1209 (93%)	419 (97%)	1.0	1.0	ref	ref
Yes	50 (4%)	12 (3%)	0.7	0.7	0.4	1.3
Missing	43 (3%)	3 (1%)	-	-	-	-
Inhalation corticosteroids						
No	1243 (96%)	418 (96%)	1.0	1.0	ref	ref
Yes	14 (1%)	8 (2%)	1.5	1.6	0.7	4.0
Missing	45 (4%)	8 (2%)	-	-	-	-
Anti-epileptic medication						
No	1255 (96%)	430 (99%)	1.0	1.0	ref	ref
Yes	7 (1%)	1 (0%)	0.4	^	^	^
Missing	40 (3%)	3 (1%)	-	-	-	-
Smoking						
No smoking	1080 (83%)	348 (80%)	1.0	1.0	ref	ref
Smoking etiological period^b^	116 (9%)	50 (12%)	**1.4**	**1.4**	**1.0**	**2.0**
Smoking other period	93 (7%)	31 (7%)	1.0	1.1	0.7	1.7
Missing	13 (1%)	5 (1%)	-	-	-	-
Alcohol						
No alcohol	646 (50%)	249 (57%)	1.0	1.0	ref	ref
Alcohol etiological period^b^	102 (8%)	29 (7%)	0.7	0.7	0.5	1.2
Alcohol other period	533 (41%)	151 (35%)	**0.7**	**0.7**	**0.6**	**0.9**
Missing	21 (2%)	5 (1%)	-	-	-	-
Folic acid supplementation						
No supplementation	153 (12%)	59 (15%)	1.0	1.0	ref	ref
Use as recommended^c^	708 (57%)	192 (47%)	**0.7**	**0.7**	**0.5**	**1.0**
Suboptimal use^d^	384 (31%)	155 (38%)	1.1	1.0	0.7	1.5
Missing			-	-	-	-
Maternal diet*						
No diet	384 (95%)	75 (93%)	1.0	1.0	ref	ref
Vegetarian	8 (2%)	2 (2%)	1.4	^	^	^
Low salt	9 (2%)	2 (2%)	1.4	^	^	^
Missing	4 (1%)	2 (2%)	-	-	-	-
Maternal stress*						
No	310 (77%)	51 (63%)	1.0	1.0	ref	ref
Yes	90 (22%)	29 (36%)	**1.9**	**2.1**	**1.2**	**3.5**
Missing	5 (1%)	1 (1%)	-	-	-	-
Family history of CAKUT						
No	1070 (82%)	330 (76%)	1.0	1.0	ref	ref
Yes	13 (1%)	32 (7%)	**12.1**	**6.6**	**2.9**	**15.1**
Missing	219 (17%)	72 (17%)	-	-	-	-
Paternal age						
≤ 24 year	24 (2%)	9 (2%)	1.2	0.7	0.3	1.8
25–29 year	185 (14%)	58 (13%)	1.0	1.0	0.7	1.4
30–34 year	471 (36%)	163 (38%)	1.0	1.0	ref	ref
35–39 year	340 (26%)	117 (27%)	1.0	0.9	0.7	1.2
≥ 40 year	152 (12%)	42 (10%)	0.8	0.8	0.5	1.2
Missing	130 (10%)	45 (10%)	-	-	-	-

^*^Assessed in full questionnaires only (*n* = 486). ^Not calculated since less than 5 cases were exposed. ^a^Adjusted for minimal set of confounders determined using directed acyclic graphs (DAGs; available in supplementary materials). ^b^Use during the etiological period was defined as exposure from the 6th week of pregnancy onwards. ^c^Use as recommended is initiation before pregnancy and continued use through at least the 8th week of pregnancy. ^d^Suboptimal use was defined as usage during only part of the recommended period. Bold values indicate associations with a 95% CI not including 1.0

*cOR*, crude odds ratio; *aOR*, adjusted odds ratio; *CI*, confidence interval; ref reference; *BMI*, body mass index; *ART*, artificial reproductive technique; *IUI*, intrauterine insemination; *IVF*, in vitro fertilization; *ICSI*, intracytoplasmic sperm injection; *CAKUT*, congenital anomalies of the kidney and urinary tract

Detailed analyses of folic acid supplementation showed that the type of supplement (i.e., folic acid only or folic acid-containing multivitamins) did not influence the effect size substantially (Table 3), in contrast to earlier studies by our group [16]. Limiting our analyses to prenatal multivitamins only did not change these results (Supplementary Table 2). Lastly, sensitivity analyses showed that the results of multivariable analyses were generally robust even in case of missingness not at random (Supplementary Table 3).

**Table 3:** Comparison of the effect of folic acid supplements and folic acid-containing multivitamins in the prevention of congenital solitary functioning kidney

	Controls (***n*** = 1302)	Cases (***n*** = 434)	cOR	aOR^a^	95% CI low	95% CI high
No vitamins at all	153 (12%)	59 (15%)	1.0	1.0	ref	ref
Any use^b^						
Folic acid only	599 (48%)	191 (47%)	0.8	0.8	0.6	1.2
Multivitamins only	68 (6%)	21 (5%)	0.8	0.8	0.4	1.6
Both	425 (34%)	135 (33%)	0.8	0.8	0.5	1.2
Use as recommended^c^						
Folic acid only	462 (37%)	133 (33%)	0.7	0.7	0.5	1.1
Multivitamins only	60 (5%)	13 (3%)	0.5	0.6	0.3	1.2
Both	186 (15%)	46 (11%)	**0.6**	**0.6**	**0.4**	**1.0**
Suboptimal use^d^						
Folic acid only	251 (20%)	88 (22%)	0.9	0.9	0.6	1.3
Multivitamins only	31 (3%)	14 (3%)	1.2	1.2	0.6	2.8
Both	102 (8%)	53 (13%)	1.4	1.3	0.8	2.2

^a^Adjusted for minimal set of confounders determined using directed acyclic graphs (DAGs; available in supplementary materials). ^b^Defined as use as recommended or suboptimal use. ^c^Defined as initiation before pregnancy and continued use through at least the 8th week of pregnancy. ^d^Usage during only part of the recommended period. Bold values indicate associations with a 95% CI not including 1.0

*cOR*, crude odds ratio; *aOR*, adjusted odds ratio; *CI*, confidence interval; *ref*, reference

## Discussion

In this study of 434 CSFK patients and 1302 matched population-based controls, we identified several environmental and parental risk factors for CSFK development. We newly identified maternal stress as a risk factor for CSFK, and confirmed previously reported associations with conception using IVF/ICSI, maternal infections during pregnancy, smoking during pregnancy, and a positive family history of CAKUT. The use of folic acid supplements and a younger maternal age seemed to reduce the risk of CSFK in our study, whereas previously reported associations with diabetes and obesity could not be confirmed.

Maternal stress during pregnancy is reported in the literature by up to 75% of women and has been associated with a range of adverse child-related outcomes, such as preterm birth, congenital heart defects, and even reduced reproductive function in the offspring later in life [26–28]. Although an association between maternal stress during pregnancy and CAKUT seems biologically plausible, we were unable to identify studies investigating this relationship. One study included urinary tract anomalies as an outcome after depression without medication use during pregnancy and found no increased prevalence of these anomalies [29]. There may be differences, however, between acute stress and chronic stress or depression. Stress is mainly thought to exhibit its negative effects via an increased production of corticosteroids, which are known to interfere with kidney development [30] and result in lower nephron endowment [31]. Although natural glucocorticosteroids are more efficiently broken down by the 11β-hydroxysteroid dehydrogenase type 2 enzyme in the placenta than synthetic variants [30], chronic stress has been shown to reduce the activity of this enzyme, thereby exposing the fetus to higher levels of cortisol [32]. As such, acute stress on top of chronic stress may be especially damaging for the fetus. Many difficulties exist when studying adverse outcomes related to stress in pregnancy: stress caused by different sources may influence outcomes differently [28], differences in maternal resilience and social support may interact with the likelihood of certain outcomes [26], and the amount of stress may be difficult to quantify using objective measurements. In our study, we defined stress based on self-reported data. This has the advantage of taking the perception of stress into account, rather than relying on the assumption that certain life events cause stress, but carries a risk of recall bias. Although we investigated stress in the first 2 months of pregnancy separately to avoid reverse causation, recall bias cannot be ruled out since questionnaires were filled out after birth.

In this study, we found that conception using IVF/ICSI was likely to be associated with an increased risk of CSFK. In a previous study by our group, with an overlap of 40 patients and 677 controls, we also found a possible association between IVF or ICSI and CAKUT in 562 CAKUT patients and 2139 controls [16]. Additionally, conception using ART resulted in a slightly elevated risk of urogenital abnormalities (OR 1.3, 95% CI 1.0–1.6) in an Australian registry study, but no subdivision into specific techniques was reported [15]. In two other large studies, an increased prevalence of birth defects was observed in children born after ART, but they did not investigate CAKUT specifically [33, 34]. The effect size found in our study, however, was comparable to the effect sizes in these studies, supporting our findings.

Associations between maternal febrile illnesses and congenital kidney malformations and bilateral kidney agenesis have been reported by two previous studies [13, 14]. In our study, we found an association with fever or other illnesses, but not with urinary tract infections. This may be explained by the amount of inflammation, which could interfere with kidney development and is usually low in lower urinary tract infections [35]. Our results also suggest that continued smoking during the etiological period may be a risk factor for CSFK, which is in line with a recent meta-analysis [12]. The fact that a family history of CAKUT was the strongest risk factor found in our study supports the hypothesis of a multifactorial etiology in which genetic factors are also involved.

Surprisingly, we did not find an association between maternal overweight or obesity and the risk of CSFK. Several other studies, including a large meta-analysis, reported a mild to moderately elevated risk of CAKUT in mothers with a high BMI [10, 36–38]. The effect estimate for obesity that we observed, however, is similar to that of the very small risk identified in the meta-analysis, but our confidence interval was much larger because of fewer participants in our study. It is hypothesized that the effect of maternal weight is mainly a consequence of higher glucose levels transmitted to the developing fetus [39]. Because undiagnosed hyperglycemia is probably less likely in The Netherlands compared to countries with less accessible healthcare systems, others may also have found larger effect sizes. Similarly, we did not observe associations for preexisting and gestational diabetes, which is partly due to small numbers but may also reflect stricter control of blood glucose in The Netherlands or our inability to select only mothers with gestational diabetes present during the etiologically relevant time period. Interestingly, the analyses for women with underweight point in the direction of an increased risk of CSFK, which is as yet unexplained. Lastly, we found a protective effect of alcohol consumption, even when studying continued drinking in pregnancy. It seems unlikely that alcohol reduces the risk of CSFK, however, especially since several others found no effect or increased ORs [9, 11, 16, 38].

One of the most successful preventive measures against congenital malformations so far is the use of folic acid supplements. Although primarily advised for the prevention of neural tube defects, lower numbers of CAKUT in children of mothers who used folic acid supplements have also been found [17, 18]. In a previous study by our group, we observed a difference in effect on CAKUT between folic acid supplements and folic acid-containing multivitamins [16]. In the current study, which was larger and focused specifically on CSFK, the effect was similar for both types of supplements (aORs 0.7 and 0.6 for folic acid supplements and folic acid-containing multivitamins, respectively). This reaffirms the recommendation given to all Dutch women who want to become pregnant to use folic acid and vitamin D supplements consistently, and to use other vitamins or supplements only on indication [40].

Maternal age was weakly associated with the risk of CSFK in our study, with an aOR of 0.8 (95% CI 0.6–1.0) for mothers between 25 and 30 years old versus mothers of 30–35 years old. Results in the literature were inconsistent: whereas Parikh et al. found higher risks of UKA for mothers < 18 years of age [9], Tain et al. reported a higher risk of CAKUT in mothers aged 20–29, 30–39, or > 40 years, compared to < 20 years [41]. In two other studies, no statistically significant results were found [42, 43]. Therefore, if an effect of maternal age exists, it is likely to be small.

Similar to many studies investigating environmental and parental causes of congenital malformations, our study was designed as a case–control study, with the inherent potential limitation of selection bias and recall bias due to its retrospective nature and the long time between childbirth and completion of the questionnaire. To reduce the possibility of selection bias, we randomly selected the majority of controls from the geographical areas where the cases came from. In addition, we matched cases and controls conditional on year of birth to rule out selection bias or confounding by year of childbirth. We believe that the effect of recall bias on our exposures is likely to be limited as well, since most factors studied represent important lifestyle habits, chronic conditions, or events that are not easily forgotten, especially not in the well-remembered period of pregnancy. If recall bias did occur, it was most likely nondifferential leading to a slight underestimation of the effect estimates (i.e., bias towards no effect), since the time to completion of the questionnaire was comparable among mothers of cases and controls. If parents of cases would have underreported exposure due to, for instance, feelings of guilt, differential bias would have occurred, also resulting in underestimation of effect estimates. Our analyses were also hampered by missing values, although this was limited to ≤ 10% for all variables except family history. To facilitate analyses of all participants, even in case of missing data, multiple imputation was used under the assumption of missingness at random. For BMI, this assumption was supported by a comparison with population data. Although we cannot exclude with absolute certainty that some information may have been missing not at random, most results proved stable when simulating such patterns of missingness. Our study benefitted from the large number of patients with a well-defined phenotype, in contrast to others who often studied the entire CAKUT spectrum. Still, etiological differences between UKA and MCDK cannot be ruled out, and the degree of association may vary between these diagnoses. In addition, information on many potential risk factors and confounders was available, and DAGs were created for each prespecified risk factor in order to correct for confounding in the most optimal fashion. No relevant unmeasured confounders were identified by the DAGs. Nonetheless, several lines of evidence are needed before a causal relationship can be established using observational data, so our results should be supported by others before they can be firmly established.

Our results reaffirm the positive effects of folic acid supplementation in pregnancy and suggest that the form in which folic acid is taken does not influence effectiveness. Furthermore, we again highlighted the potential harmful effects of smoking and identified stress as a risk factor for CSFK. The identification of stress as a novel risk factor warrants reexamination by others, but given the well-established range of negative effects of stress during pregnancy, stress reduction strategies could already be incorporated into public health advice. Several non-pharmacological measures that improve coping and reduce potential negative effects of stress during pregnancy are available.

As illustrated by the current study, the etiology of CSFK is likely to consist of a combination of genetic and environmental factors. Although both factors may cause CSFK independently, interactions are likely to play a role, given the low number of genetically solved cases and the relatively modest effect sizes of most environmental factors. Nevertheless, only a few successful gene–environment interaction studies are available. For cleft lip/palate, one of the most common birth defects, investigators found evidence of an interaction between genetic variants and both multivitamin supplements and exposure to environmental tobacco smoke [44]. We are not aware of any studies that have investigated whether similar interactions play a role in kidney development, however, but large cohorts integrating genetic and environmental data are needed to do so in the future.

In conclusion, we show that several environmental and parental risk factors are likely to play a role in the etiology of CSFK. Government agencies and healthcare workers should focus on optimizing lifestyle and other targetable factors in women wanting to become pregnant, while further studies should incorporate environmental factors when studying the etiology of CSFK.

## Supplementary Information

Below is the link to the electronic supplementary material.

Supplementary file1 (DOCX 456 KB)

Graphical Abstract (PPTX 78 KB)

## Data Availability

The datasets generated during and/or analyzed during the current study are available from the corresponding author on reasonable request. The questionnaire that was used to obtain data on the potential risk factors is shared as supplementary material and available on the website https://www.agoraproject.nl/
