# Supplementary material for: Environmental and parental risk factors for congenital solitary functioning kidney — a case–control study
Source: Pediatr Nephrol. 2023 Feb 20;38(8):2631–41. doi: 10.1007/s00467-023-05900-6 (PMC10393837; doi:10.1007/s00467-023-05900-6)
Supplement: Supplementary file 1 — Supplementary file1 (DOCX 456 KB) [file 467_2023_5900_MOESM1_ESM.docx]

# Environmental and parental risk factors for congenital solitary functioning kidney – a case-control study

Sander Groen in ‘t Woud MD^1,2^, Nel Roeleveld PhD^1^, Iris A.L.M. van Rooij PhD^1^, Wout F.J. Feitz MD PhD^3^, Michiel F. Schreuder MD PhD^2*^, and Loes F.M. van der Zanden PhD^1*^ for the SOFIA study

^1^ Radboud university medical center, Department for Health Evidence, Nijmegen, The Netherlands

^2^ Radboudumc Amalia Children’s Hospital, Department of Pediatric Nephrology, Nijmegen, The Netherlands

^3^ Radboudumc Amalia Children’s Hospital, Department of Urology, Division of Pediatric Urology, Nijmegen, The Netherlands

* Authors contributed equally

**Corresponding author:**

Loes van der Zanden

Radboud university medical center

Department for Health Evidence

P.O. Box 9101

6500 HB Nijmegen

The Netherlands

Loes.vanderzanden@radboudumc.nl

T: +31 629690111

F: +31 24 3616428

**Supplementary Table 1** Clinical characteristics of 434 children with a congenital solitary functioning kidney included in the study

|  | | N (%) |
| --- | --- | --- |
| Sex |  | |
| Male | 283 (65%) | |
| Female | 151 (35%) | |
| Cause of the CSFK |  | |
| Unilateral kidney agenesis | 151 (35%) | |
| Multicystic dysplastic kidney | 283 (65%) | |
| Side of the CSFK |  | |
| Left | 205 (47%) | |
| Right | 228 (53%) | |
| Missing | 1 (0%) | |
| Antenatal diagnosis |  | |
| Yes | 363 (84%) | |
| No | 26 (6%) | |
| Missing | 45 (10%) | |
| Additional CAKUT in the CSFK |  | |
| Yes | 134 (31%) | |
| No | 238 (55%) | |
| Missing | 62 (14%) | |
| Extrarenal congenital anomalies |  | |
| Yes | 102 (24%) | |
| No | 309 (71%) | |
| Missing | 23 (5%) | |
| Gestational age at childbirth |  | |
| Term birth (≥37 weeks) | 366 (84%) | |
| Preterm birth (<37 weeks) | 42 (10%) | |
| Missing | 26 (6%) | |
| Birthweight |  | |
| Normal birthweight (≥2500 gram) | 354 (82%) | |
| Low birthweight (<2500 gram) | 51 (12%) | |
| Missing | 29 (7%) | |

CSFK congenital solitary functioning kidney, CAKUT congenital anomalies of the kidney and urinary tract

**Supplementary Table 2** Comparison of the effect of folic-acid supplements and folic-acid containing multivitamins in prevention of congenital solitary functioning kidney, limited to prenatal multivitamins.

|  | Controls  (n = 1302) | Cases  (n = 434) | cOR | aOR^a^ | 95% CI low | 95% CI high |
| --- | --- | --- | --- | --- | --- | --- |
| No vitamins at all | 153 (12%) | 59 (15%) | 1.0 | 1.0 | ref | ref |
| Any use^b^ |  |  |  |  |  |  |
| Folic acid only | 620 (50%) | 202 (50%) | 0.8 | 0.8 | 0.6 | 1.2 |
| Multivitamins only | 58 (5%) | 20 (5%) | 0.8 | 0.9 | 0.5 | 1.8 |
| Both | 404 (33%) | 124 (31%) | 0.8 | 0.8 | 0.5 | 1.2 |
| Use as recommended^c^ |  |  |  |  |  |  |
| Folic acid only | 471 (38%) | 136 (34%) | 0.7 | 0.7 | 0.5 | 1.1 |
| Multivitamins only | 48 (4%) | 8 (2%) | 0.4 | **0.4** | **0.2** | **1.0** |
| Both | 177 (14%) | 43 (11%) | **0.6** | **0.6** | **0.4** | **1.0** |
| Suboptimal use^d^ |  |  |  |  |  |  |
| Folic acid only | 255 (21%) | 91 (23%) | 0.9 | 0.9 | 0.6 | 1.4 |
| Multivitamins only | 27 (2%) | 14 (4%) | 1.3 | 1.4 | 0.6 | 3.2 |
| Both | 98 (8%) | 50 (13%) | 1.4 | 1.3 | 0.8 | 2.2 |

^a^Adjusted for minimal set of confounders determined using directed acyclic graphs (DAGs; available as supplementary table 2 and supplementary figure 1). ^b^Defined as use as recommended or suboptimal use. ^c^Defined as initiation before pregnancy and continued use through at least the 8^th^ week of pregnancy. ^d^Usage during only part of the recommended period.

cOR crude odds ratio, aOR adjusted odds ratio, CI confidence interval, ref reference

**Supplementary Table 3** Sensitivity analyses comparing analyses of the imputed database under the assumption of missingness at random with analyses under two scenarios of missingness not at random (*i.e.* all missing values classified in the reference category and all missing values classified as exposed).

|  | Imputed | | | Missing = reference | | | Missing = abnormal^a^ | | |
| --- | --- | --- | --- | --- | --- | --- | --- | --- | --- |
|  | aOR^b^ | 95% CI low | 95% CI high | aOR^b^ | 95% CI low | 95% CI high | aOR^b^ | 95% CI low | 95% CI high |
| Gravidity |  |  |  |  |  |  |  |  |  |
| First pregnancy | 1.0 | ref | ref | 1.0 | ref | ref | 1.0 | ref | ref |
| Subsequent pregnancy | 1.0 | 0.8 | 1.2 | 0.9 | 0.7 | 1.2 | 1.0 | 0.8 | 1.2 |
| Season of conception |  |  |  |  |  |  |  |  |  |
| Spring | 1.0 | ref | ref | 1.0 | ref | ref | 1.0 | ref | ref |
| Summer | 1.2 | 0.9 | 1.6 | 1.2 | 0.8 | 1.6 | 1.2 | 0.9 | 1.6 |
| Fall | 1.1 | 0.8 | 1.5 | 1.1 | 0.8 | 1.5 | 1.1 | 0.8 | 1.5 |
| Winter | 1.0 | 0.7 | 1.4 | 1.0 | 0.7 | 1.3 | 1.0 | 0.8 | 1.4 |
| Maternal age |  |  |  |  |  |  |  |  |  |
| =< 24 year | 1.2 | 0.6 | 2.2 | 1.1 | 0.6 | 2.0 | 1.1 | 0.6 | 2.0 |
| 25-29 year | **0.8** | **0.6** | **1.0** | **0.7** | **0.6** | **1.0** | **0.8** | **0.6** | **1.0** |
| 30-34 year | 1.0 | ref | ref | 1.0 | ref | ref | 1.0 | ref | ref |
| 35-39 year | 1.1 | 0.8 | 1.5 | 1.1 | 0.8 | 1.5 | 1.1 | 0.8 | 1.5 |
| >= 40 year | 0.5 | 0.2 | 1.3 | 0.5 | 0.2 | 1.3 | 0.9 | 0.5 | 1.7 |
| Maternal BMI |  |  |  |  |  |  |  |  |  |
| Underweight (<18.5 kg/m2) | 1.6 | 0.9 | 2.9 | **1.8** | **1.0** | **3.1** | 1.4 | 0.7 | 3.0 |
| Normal (18.5-24.9 kg/m2) | 1.0 | ref | ref | 1.0 | ref | ref | 1.0 | ref | ref |
| Overweight (25-29.9 kg/m2) | 1.1 | 0.9 | 1.5 | 1.1 | 0.9 | 1.5 | 1.1 | 0.8 | 1.6 |
| Obese (>=30 kg/m2) | 1.1 | 0.7 | 1.8 | 1.2 | 0.8 | 1.8 | 1.2 | 0.8 | 2.0 |
| Subfertility |  |  |  |  |  |  |  |  |  |
| Fertile | 1.0 | ref | ref | 1.0 | ref | ref | 1.0 | ref | ref |
| Subfertile without ART | 1.0 | 0.7 | 1.4 | 1.1 | 0.8 | 1.5 | 1.0 | 0.7 | 1.4 |
| IUI without hormones | 0.8 | 0.3 | 2.4 | 1.0 | 0.4 | 2.7 | 0.9 | 0.3 | 2.4 |
| Hormonal without IVF/ICSI | 0.7 | 0.3 | 1.5 | 0.8 | 0.4 | 1.7 | 0.7 | 0.3 | 1.5 |
| IVF/ICSI | **1.8** | **1.0** | **3.2** | **1.8** | **1.0** | **3.3** | **0.5** | **0.4** | **0.8** |
| Maternal diabetes |  |  |  |  |  |  |  |  |  |
| No diabetes | 1.0 | ref | ref | 1.0 | ref | ref | 1.0 | ref | ref |
| Preexisting diabetes | ^ | ^ | ^ | ^ | ^ | ^ | ^ | ^ | ^ |
| Gestational diabetes | 1.1 | 0.5 | 2.2 | 1.0 | 0.5 | 2.1 | **0.3** | **0.2** | **0.7** |
| Preexisting hypertension |  |  |  |  |  |  |  |  |  |
| No | 1.0 | ref | ref | 1.0 | ref | ref | 1.0 | ref | ref |
| Yes | 0.7 | 0.3 | 1.8 | 0.7 | 0.3 | 1.8 | 0.7 | 0.3 | 1.6 |
| Maternal infections* |  |  |  |  |  |  |  |  |  |
| No infection | 1.0 | ref | ref | 1.0 | ref | ref | 1.0 | ref | ref |
| Cystitis | 1.5 | 0.6 | 3.6 | 1.2 | 0.5 | 3.1 | 0.9 | 0.4 | 2.4 |
| Other infection/fever | **2.5** | **1.4** | **4.7** | **2.5** | **1.3** | **4.8** | **2.2** | **1.2** | **4.1** |
| Anti-diabetic medication |  |  |  |  |  |  |  |  |  |
| No | 1.0 | ref | ref | 1.0 | ref | ref | 1.0 | ref | ref |
| Yes | 1.7 | 0.4 | 7.6 | 1.9 | 0.4 | 8.3 | 0.6 | 0.2 | 1.7 |
| Anti-hypertensive medication |  |  |  |  |  |  |  |  |  |
| No | 1.0 | ref | ref | 1.0 | ref | ref | 1.0 | ref | ref |
| Yes | 0.7 | 0.4 | 1.3 | 0.7 | 0.4 | 1.3 | 0.5 | 0.3 | 0.8 |
| Inhalation corticosteroids |  |  |  |  |  |  |  |  |  |
| No | 1.0 | ref | ref | 1.0 | ref | ref | 1.0 | ref | ref |
| Yes | 1.6 | 0.7 | 4.0 | 1.7 | 0.7 | 4.1 | 0.9 | 0.5 | 1.6 |
| Anti-epileptic medication |  |  |  |  |  |  |  |  |  |
| No | 1.0 | ref | ref | 1.0 | ref | ref | 1.0 | ref | ref |
| Yes | ^ | ^ | ^ | ^ | ^ | ^ | ^ | ^ | ^ |
| Smoking |  |  |  |  |  |  |  |  |  |
| No smoking | 1.0 | ref | ref | 1.0 | ref | ref | 1.0 | ref | ref |
| Smoking etiological period^c^ | **1.4** | **1.0** | **2.0** | **1.5** | **1.0** | **2.1** | 1.1 | 0.8 | 1.7 |
| Smoking other period | 1.1 | 0.7 | 1.7 | 1.1 | 0.7 | 1.6 | 1.0 | 0.6 | 1.7 |
| Alcohol |  |  |  |  |  |  |  |  |  |
| No alcohol | 1.0 | ref | ref | 1.0 | ref | ref | 1.0 | ref | ref |
| Alcohol etiological period^c^ | 0.7 | 0.5 | 1.2 | 0.8 | 0.5 | 1.2 | **0.6** | **0.4** | **0.9** |
| Alcohol other period | **0.7** | **0.6** | **0.9** | **0.8** | **0.6** | **1.0** | **0.7** | **0.5** | **0.9** |
| Folic acid supplementation |  |  |  |  |  |  |  |  |  |
| No supplementation | 1.0 | ref | ref | 1.0 | ref | ref | 1.0 | ref | ref |
| Use as recommended^d^ | **0.7** | **0.5** | **1.0** | **0.7** | **0.5** | **0.9** | 0.8 | 0.5 | 1.1 |
| Suboptimal use^e^ | 1.0 | 0.7 | 1.5 | 1.0 | 0.7 | 1.4 | 1.1 | 0.7 | 1.5 |
| Maternal diet* |  |  |  |  |  |  |  |  |  |
| No diet | 1.0 | ref | ref | 1.0 | ref | ref | 1.0 | ref | ref |
| Vegetarian | ^ | ^ | ^ | ^ | ^ | ^ | ^ | ^ | ^ |
| Low salt | ^ | ^ | ^ | ^ | ^ | ^ | ^ | ^ | ^ |
| Maternal stress* |  |  |  |  |  |  |  |  |  |
| No | 1.0 | ref | ref | 1.0 | ref | ref | 1.0 | ref | ref |
| Yes | **2.1** | **1.2** | **3.5** | **2.0** | **1.2** | **3.4** | **2.0** | **1.2** | **3.3** |
| Family history of CAKUT |  |  |  |  |  |  |  |  |  |
| No | 1.0 | ref | ref | 1.0 | ref | ref | 1.0 | ref | ref |
| Yes | **6.6** | **2.9** | **15.1** | **7.9** | **4.1** | **15.2** | **1.5** | **1.1** | **1.9** |
| Paternal age |  |  |  |  |  |  |  |  |  |
| =< 24 year | 0.7 | 0.3 | 1.8 | 0.9 | 0.4 | 2.2 | 0.8 | 0.3 | 2.0 |
| 25-29 year | 1.0 | 0.7 | 1.4 | 1.0 | 0.7 | 1.4 | 1.0 | 0.7 | 1.4 |
| 30-34 year | 1.0 | ref | ref | 1.0 | ref | ref | 1.0 | ref | ref |
| 35-39 year | 0.9 | 0.7 | 1.2 | 0.9 | 0.7 | 1.2 | 0.9 | 0.7 | 1.2 |
| >= 40 year | 0.8 | 0.5 | 1.2 | 0.8 | 0.5 | 1.2 | 0.8 | 0.6 | 1.1 |

*Assessed in paper questionnaires only (n = 486). ^Not calculated since less than 5 cases were exposed. ^a^In case of several exposed categories, the most extreme value was taken (*e.g.* for fertility exposure to IVF/ICSI and for smoking exposure in the etiological period). ^b^Adjusted for minimal set of confounders determined using directed acyclic graphs (DAGs; available in supplementary materials). ^c^Use during the etiological period was defined as exposure from the 6th week of pregnancy onwards. ^d^Use as recommended is initiation before pregnancy and continued use through at least the 8^th^ week of pregnancy. ^e^Suboptimal use was defined as usage during only part of the recommended period.

aOR adjusted odds ratio, CI confidence interval, ref reference, BMI body mass index, ART artificial reproductive technique, IUI intrauterine insemination, IVF in vitro fertilization, ICSI intracytoplasmic sperm injection, CAKUT congenital anomalies of the kidney and urinary tract

**Supplementary Table 4** Minimal set of confounders obtained from directed acyclic graphs.

| **Exposure** | **Minimal set of confounders** |
| --- | --- |
| Gravidity | Maternal ethnicity, education level, and age |
| Season of conception | No adjustment needed |
| Maternal age | Year of childbirth, maternal education level, and paternal age |
| Maternal BMI | Year of childbirth, gravidity, season of conception, maternal ethnicity, education level, age, alcohol use, smoking, and diet, |
| Subfertility | Year of childbirth, gravidity, season of conception, maternal ethnicity, education level, age, BMI, and smoking, and paternal age |
| Preexisting diabetes | Maternal ethnicity and age |
| Gestational diabetes | Season of conception, maternal ethnicity, education level, age, subfertility, BMI, preexisting diabetes, smoking, and folic acid supplementation, and subfertility |
| Preexisting hypertension | Year of childbirth, gravidity, season of conception, maternal ethnicity, education level, age, BMI, subfertility, preexisting diabetes, gestational diabetes, alcohol use, smoking, and folic acid supplementation |
| Maternal infections | Season of conception, maternal ethnicity, BMI, preexisting diabetes, smoking, diet, and stress |
| Medication use during pregnancy | Year of childbirth, maternal preexisting diabetes, subfertility, and infections |
| Smoking | Year of childbirth, maternal age, subfertility, smoking, and stress |
| Alcohol | Year of childbirth, maternal age, subfertility, alcohol use, and stress |
| Folic acid supplementation | Year of childbirth, Maternal ethnicity, education level, gravidity, age, subfertility, and preexisting diabetes |
| Maternal diet | Year of childbirth, maternal ethnicity, education level, and preexisting diabetes |
| Maternal stress | Maternal education level |
| Family history | Maternal ethnicity |
| Paternal age | Year of childbirth, maternal education level, and age |


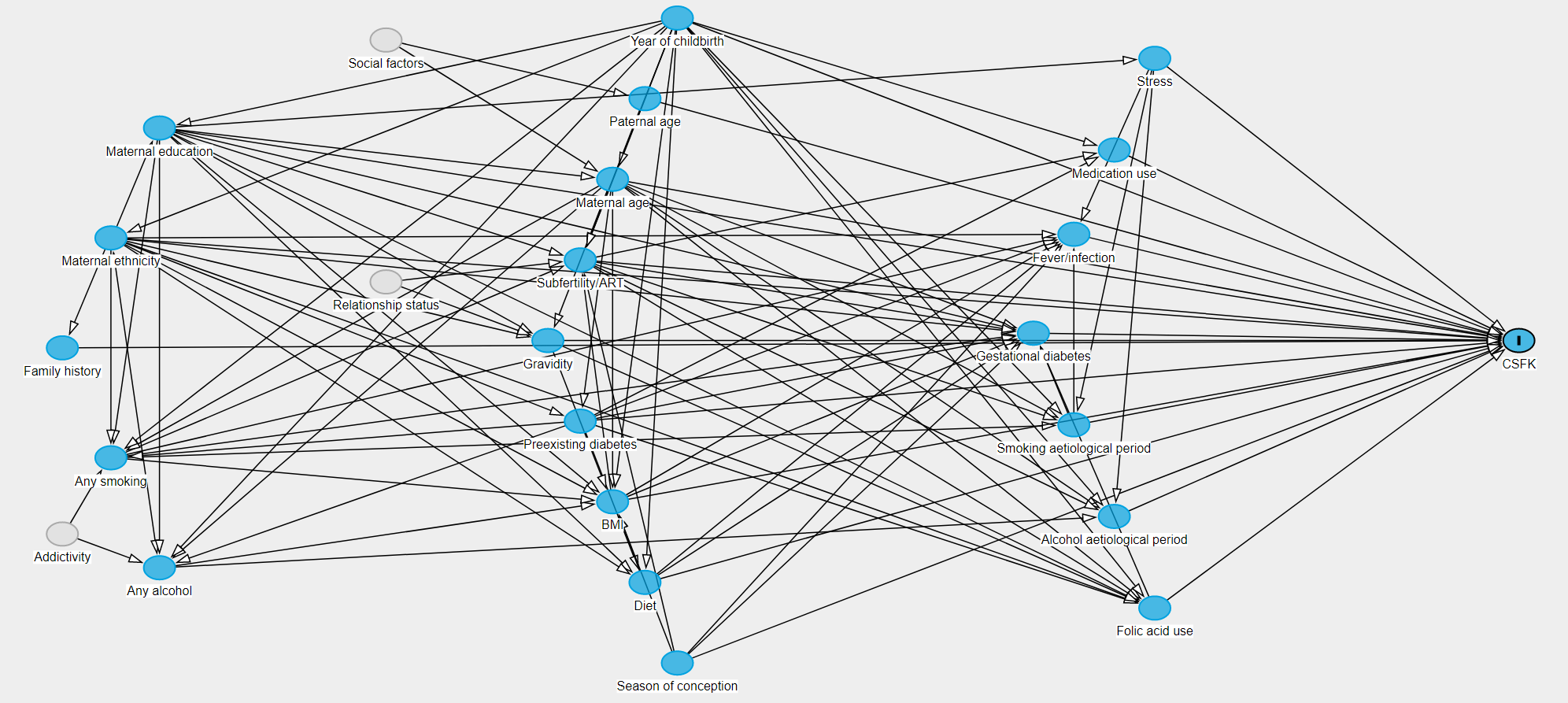


**Supplementary Figure 1** Directed Acyclic Graph containing all potential risk factors and confounders with their mutual relations. Factors with grey circles represent unobserved variables.

BMI body mass index, CSFK congenital solitary functioning kidney

**Supplementary Figure 2** Matrix containing all associations between potential risk factors and confounders. Double arrows (<- ->) indicate a shared unobserved parent variable.
