# Supplementary material for: Environmental and parental risk factors for congenital solitary functioning kidney — a case–control study
Source: Pediatr Nephrol. 2023 Feb 20;38(8):2631–41. doi: 10.1007/s00467-023-05900-6 (PMC10393837; doi:10.1007/s00467-023-05900-6)
Supplement: Supplementary file 2 — Graphical Abstract (PPTX 78 KB) [file 467_2023_5900_MOESM2_ESM.pptx]

## Slide 1
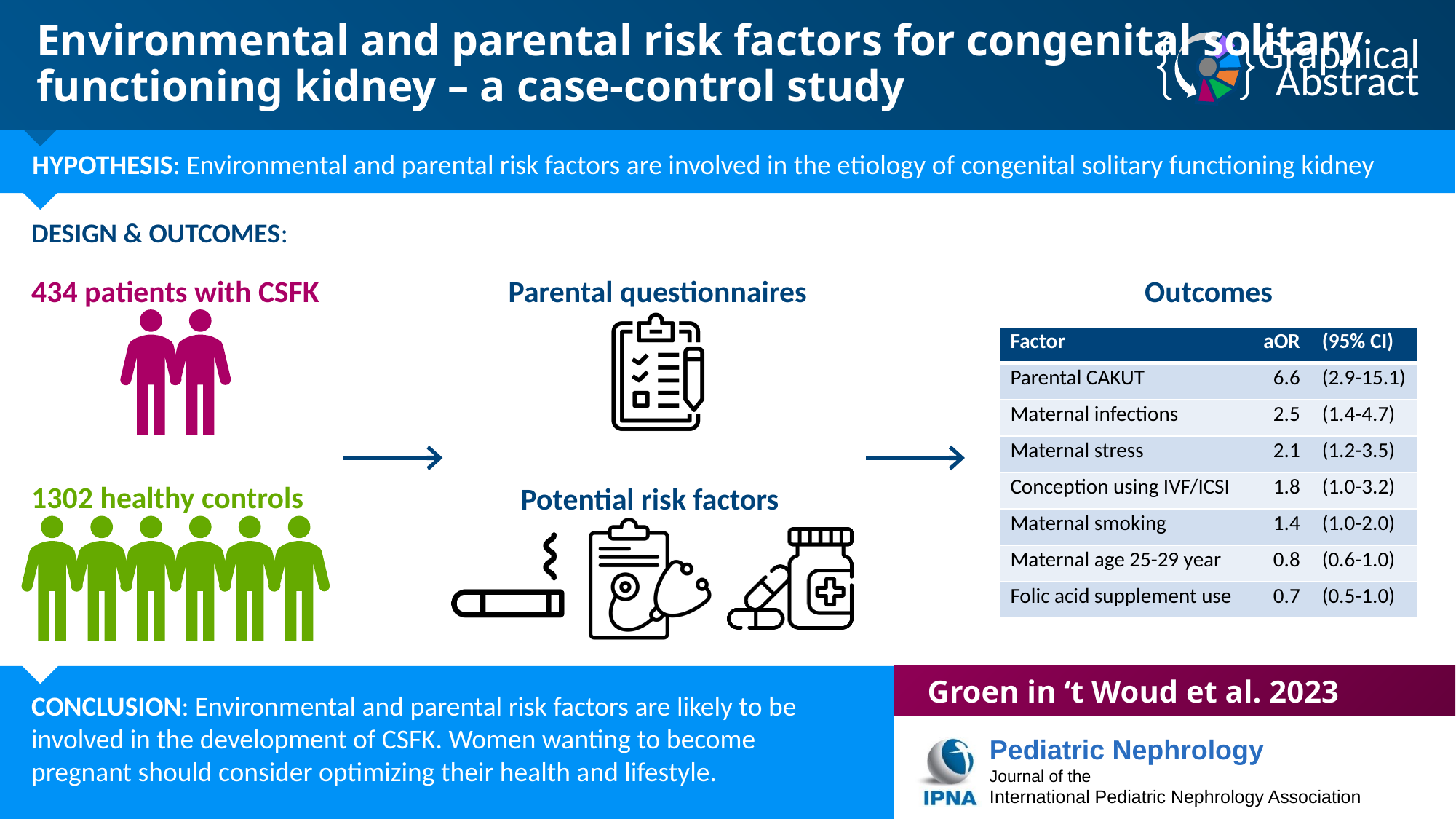

Environmental and parental risk factors for congenital solitary
functioning kidney – a case-control study
HYPOTHESIS: Environmental and parental risk factors are involved in the etiology of congenital solitary functioning kidney
DESIGN & OUTCOMES:
434 patients with CSFK
Parental questionnaires
Outcomes
| Factor | aOR | (95% CI) |
| --- | --- | --- |
| Parental CAKUT | 6.6 | (2.9-15.1) |
| Maternal infections | 2.5 | (1.4-4.7) |
| Maternal stress | 2.1 | (1.2-3.5) |
| Conception using IVF/ICSI | 1.8 | (1.0-3.2) |
| Maternal smoking | 1.4 | (1.0-2.0) |
| Maternal age 25-29 year | 0.8 | (0.6-1.0) |
| Folic acid supplement use | 0.7 | (0.5-1.0) |
1302 healthy controls
Potential risk factors
Groen in ‘t Woud et al. 2023
CONCLUSION: Environmental and parental risk factors are likely to be involved in the development of CSFK. Women wanting to become pregnant should consider optimizing their health and lifestyle.
